# Arc-arc collision caused the 2018 Eastern Iburi earthquake (*M* 6.7) in Hokkaido, Japan

**DOI:** 10.1038/s41598-019-50305-x

**Published:** 2019-09-26

**Authors:** Yuanyuan Hua, Dapeng Zhao, Yixian Xu, Zewei Wang

**Affiliations:** 1 Department of Geophysics, Graduate School of Science, Tohoku University, Sendai, 980-8578 Japan; 2 Subsurface Multi-scale Imaging Key Laboratory of Hubei Province, Institute of Geophysics and Geomatics, China University of Geosciences, Wuhan, 430074 China; 3 School of Earth Sciences, Zhejiang University, Hangzhou, Zhejiang 310027 China; 4 School of Earth Sciences and Engineering, Sun Yat-sen University, Guangzhou, China

**Keywords:** Geophysics, Seismology

## Abstract

Inland crustal earthquakes usually occur in the brittle upper crust (0–20 km depths), but the 6 September 2018 Eastern Iburi earthquake (M 6.7) took place in southern Hokkaido with a focal depth of ~37 km, causing 41 fatalities and serious damage to the local infrastructure. The reason why this event was so deep and its causal mechanism are still unclear. In this work we study the three-dimensional P and S wave seismic attenuation (1/Q) structure in the source zone of the 2018 Iburi earthquake. Our results show that this event occurred at the boundary between the Sorachi-Yezo belt (low Q) and the dipping Northeastern (NE) Japan arc (high Q) that is descending beneath the Kuril arc. The collision between the NE Japan and Kuril arcs as well as fluids from dehydration of the subducting Pacific plate caused this big event and its unusual focal depth. Similar attenuation structures are revealed in source zones of the 1970 Hidaka earthquake (M 6.7) and the 1982 Urakawa-oki earthquake (M 7.1), suggesting that they were caused by similar processes. We think that large earthquakes will take place again on the active thrust faults in southern Hokkaido in the coming decades. Hence, we should pay much attention to the seismic risk and prepare for reduction of earthquake hazards there.

## Introduction

**Figure 1:**
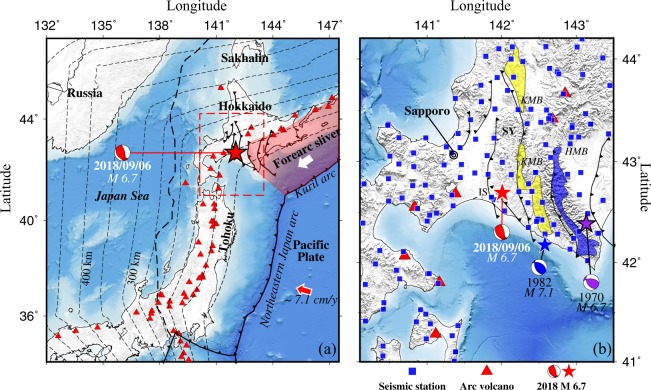
Tectonic settings and seismic stations used in this study. (**a**) Tectonic settings of the study region. The red dashed line denotes the location of the present study area. The red star and the red beach ball represent the epicenter and focal mechanism of the 2018 Eastern Iburi earthquake (M 6.7). The black solid and dashed lines show the plate boundaries. The thin dashed lines denote depth contours of the upper boundary of the subducting Pacific slab, which is modified from refs^56,59^. The pink zone shows the Kuril forearc sliver. The red triangles denote active volcanoes. (**b**) Distribution of 136 seismic stations (blue squares) used in this study. The black solid lines denote major active faults. The blue and purple stars denote epicenters of the 1982 Urakawa-oki earthquake (M 7.1) and the 1970 Hidaka earthquake (M 6.7), respectively. The yellow and blue patches denote the Kamuikatan metamorphic belt and the Hidaka metamorphic belt, respectively. This figure was generated using the Generic Mapping Tools^58^ version 5.4.3 (http://gmt.soest.hawaii.edu).

The Eastern Iburi earthquake (M 6.7 in the Japan Meteorological Agency (JMA) scale) occurred in southern Hokkaido (Fig. 1) on 6 September 2018 with a focal depth of ~ 37 km, which caused 41 fatalities and 691 injured. Its mainshock produced strong ground motions with a peak ground acceleration of up to 1796 gal^1^. Serious and widespread landslides were trigged by the mainshock and its aftershocks. To date, many large earthquakes have occurred in and around southern Hokkaido, including the 1970 Hidaka earthquake (M 6.7) and the 1982 Urakawa-Oki earthquake (M 7.1). All these large events caused serious damage to the local society and infrastructure in Hokkaido.

Earthquakes in and around Hokkaido can be divided into three types, including megathrust earthquakes at depths of ~10–50 km along the upper boundary of the subducting Pacific plate, intra-slab events that occur within the subducting Pacific slab, and earthquakes in the overriding Okhotsk plate^2^. Inland crustal earthquakes in the Okhotsk plate usually occur within the upper crust down to a depth of ~20 km (ref.^3^). Hence, the 2018 Eastern Iburi earthquake with a focal depth of ~37 km is quite unusual because it is much deeper than other inland crustal events in the region, and it seems located in the uppermost mantle, considering the Moho depth of ~33 km in the source area^4,5^. It is important to investigate the three-dimensional (3-D) structure of the source zone, which may shed new light on seismotectonics and seismogenesis of the region.

Southern Hokkaido is located at the junction between the northeastern (NE) Japan arc and the Kuril arc, which originated from the interaction of the Eurasian plate with the Okhotsk plate in the Late Eocene^6–8^ (Fig. 1a). The tectonic evolution of Hokkaido has been dominated by a series of accretions and the right-lateral oblique collision between the Kuril arc and the NE Japan arc, which have formed a west-vergent fold-thrust belt called the Hidaka collision zone and a series of nearly north-south trending geological units in central Hokkaido^6,7^ (Fig. 1b). These geological units include the Sorachi-Yezo belt, the Idonnappu belt, the Hidaka belt, the Yubetsu belt and the Tokoro belt, which are mainly caused by the southwestward migration of the Kuril forearc sliver^9^ as confirmed by GPS observations^10^ (Fig. 1b). So far, many researchers have used different methods to investigate the 3-D crustal and upper mantle structure in and around Hokkaido including the Hidaka collision zone, such as wide-angle reflection seismic soundings^6,11^, seismic velocity tomography^9,12–14^, attenuation tomography^15,16^, and P-wave anisotropic tomography^17–20^. These previous studies have improved our understanding of the arc-arc collision and seismotectonics in the region.

Seismic velocity tomography has been applied extensively to study the detailed 3-D structure in source areas of large earthquakes in Japan and other regions in the world^2^ and is proven to be a powerful technique to investigate the seismogenesis and seismotectonics. Compared with seismic velocity, seismic attenuation (expressed by reciprocal of quality factor, 1/Q) is more sensitive to material properties, such as temperature, grain size, and water content in the crust and upper mantle^21–24^. Although there have been a few studies of seismic attenuation tomography in Japan (e.g. refs^15,16,25,26^), such studies are still quite few as compared with seismic velocity tomography, in particular, in source zones of large earthquakes.

Although a 3-D seismic attenuation model under Hokkaido has been determined by ref.^16^, it is a P-wave attenuation model focusing on the relation between seismic attenuation and geological structures. In the present work, to clarify the causal mechanism of the 2018 Eastern Iburi earthquake, we determine both P and S wave attenuation (Qp, Qs) tomography of the crust and upper mantle in and around the source zone of the 2018 Iburi earthquake. We used a larger number of high-quality waveform data recorded at 136 seismic stations (Fig. 1b) from 542 local shallow and intermediate-depth earthquakes (Supplementary Fig. S1). Our results shed new light on the causal mechanism of this damaging earthquake, as well as arc-arc collision and subduction dynamics in the study region.

## Results

**Figure 2:**
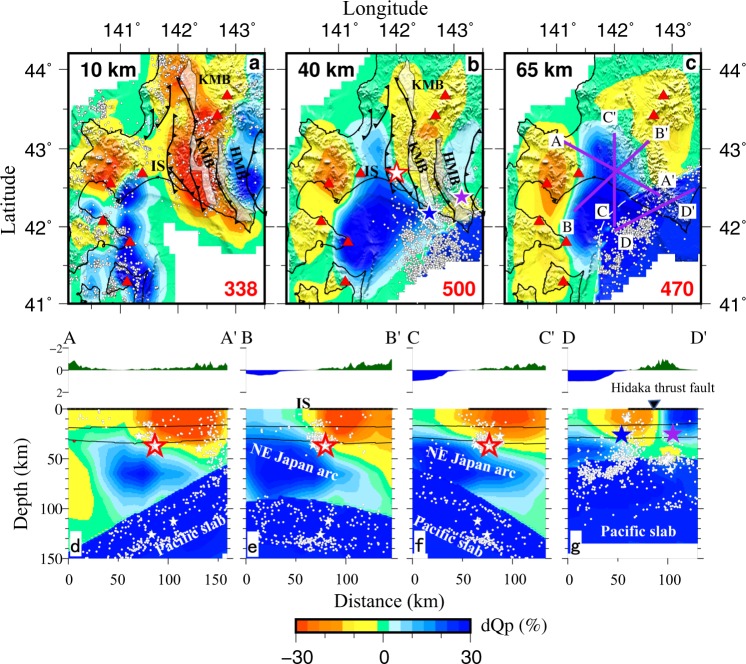
Results of P-wave attenuation (Qp) tomography. (**a**–**c**) are map views of Qp tomography at three depths. The layer depth is shown at the upper-left corner of each map. The average Qp value at each depth is shown at the lower-right corner of each map. IS, the Ishikari low land; KMB, the Kamuikatan metamorphic belt; HMB, the Hidaka metamorphic belt. The Qp perturbation (in %) scale relative to the average value is shown at the bottom. The red triangles denote active volcanoes. The white dots denote seismicity that occurred within a 2-km depth range of each layer. The black thick lines represent the major active faults. The white dashed line denotes location of the upper boundary of the subducting Pacific slab at each depth. (**d**–**g**) are vertical cross-sections of Qp tomography. Locations of the vertical cross-sections are shown in (**c**). The surface topography along each profile is shown above the cross-section. The white dots denote the background seismicity (M >2.0) during 2004–2018 within a 20-km width of each profile. The white stars denote large earthquakes (M >5.0) that occurred within a 20-km width of each profile during 2004–2018. The three thin black lines in each panel denote the Conrad and Moho discontinuities and the upper boundary of the subducting Pacific slab, which are modified from refs^3,4,56^. The red, blue and purple stars denote the 2018 Eastern Iburi earthquake (M 6.7), the 1982 Urakawa-oki earthquake (M 7.1) and the 1970 Hidaka earthquake (M 6.7), respectively. This figure was generated using the Generic Mapping Tools^58^ version 5.4.3 (http://gmt.soest.hawaii.edu).

**Figure 3:**
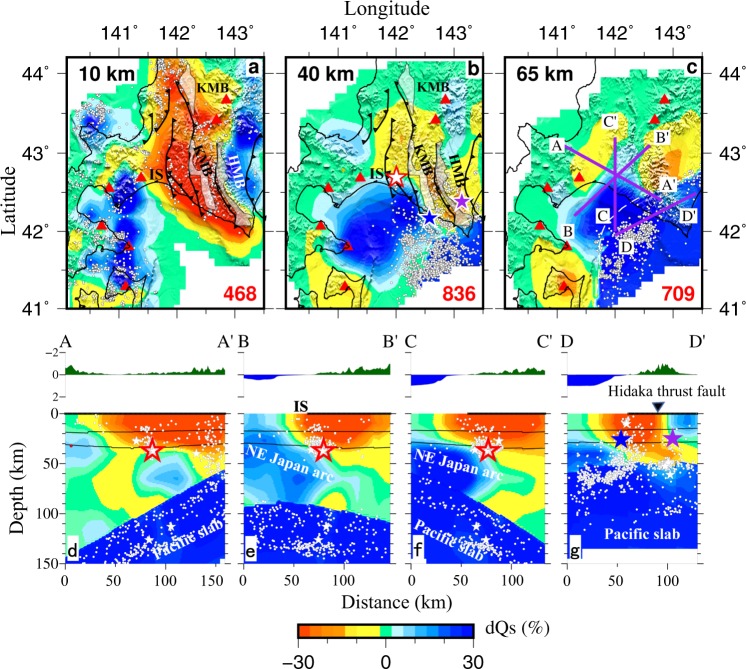
Results of S-wave attenuation (Qs) tomography. The same as Fig. 2 but for Qs tomography. This figure was generated using the Generic Mapping Tools^58^ version 5.4.3 (http://gmt.soest.hawaii.edu).

Results of Qp and Qs tomography obtained by this study are shown in Figs 2, 3. Following previous studies (e.g. refs^2,12,15,25^), the high-velocity (high-V) and low-attenuation (high-Q) Pacific slab is considered in the starting model for the tomographic inversion, so that the P and S wave ray paths can be traced more accurately. The Qp and Qs models are correlated with each other (Figs 2 and 3), though they are obtained independently. We have also conducted tomographic inversions without the predefinition of high-Q in the Pacific slab and the slab interface. The results (Supplementary Figs S2 and S3) generally show a clear high-Q slab, which are similar to the inversion results with the slab predefinition (Figs 2 and 3). Following ref.^27^, we also conducted an inversion by taking 1/Q values at the 3-D grid nodes as unknown parameters. Instead of the damped least-squares method, the L-BFGS-B algorithm^28,29^ is used to solve the system of observation equations that are associated with 1/Q. The obtained Qp and Qs results (Supplementary Fig. S4) show a pattern similar to that of Figs 2 and 3. Results of these different tomographic inversions (Supplementary Figs S2–S4) suggest that the Qp and Qs models obtained by this study (Figs 2 and 3) are quite stable and reliable.

At depths of 10 and 40 km, a clear low-Q (high attenuation) belt exists in central Hokkaido (Figs 2 and 3), which is roughly oriented in the north-south direction and corresponds to the Sorachi-Yezo (SY) belt including the Kamuikatan metamorphic (KM) belt (Fig. 1). In the east of the low-Q zone, a high-Q (low attenuation) zone exists at 10–40 km depths, which reflects the Hidaka belt. At 40 km depth, another high-Q zone is visible in the west of the low-Q zone, which reflects the Ishikari lowland. The Ishikari lowland fault system is located at the western edge of the low-Q zone (Figs 2 and 3).

Our tomographic results show a clear low-Q zone in the SY belt and high-Q zones in the Hidaka belt and Ishikari lowland at depths of 10 to 40 km (Figs 2 and 3), which are generally consistent with the previous attenuation model^16^ and velocity models of the study region (e.g. refs^9,17–19^). Our resolution tests indicate that these features are robust (Supplementary Figs S5 and S6). A velocity tomography model^9^ shows a clear low-velocity (low-V) zone in the SY belt, which corresponds to the low-Q zone imaged by this study. The SY belt is composed of three units: the Sorachi ophiolitic unit that contains the Tithonian radiolarian chert, the Yezo Supergroup of forearc basin sediments, and high P-T metamorphic rocks (the Kamuikotan metamorphic (KM) belt)^30^. Most parts of the SY belt are filled with sediments and ophiolite, which may contain a large amount of fluid and so exhibit low-Q and low-V^9^. Micro-earthquakes occur actively in the low-Q and low-V zone along the SY belt (Figs 2d and 3d), suggesting a high crack density there and highlighting a cause of the low-Q zone^16,22^. The low-Q zone in the SY belt may also reflect ascending fluids from dehydration of the Pacific slab. The ascending fluids may enter the crust that has a high crack density, leading to the low-Q anomaly.

Our results show a low-Q anomaly along the KM belt at depths of 10–40 km (Figs 2a,b and 3a,b). The KM belt is characterized by the concomitant existence of serpentinites, blueschists, metabasalts, metagabbros and metasediments of the Mesozoic age, among which serpentinite is preponderant^31^. The serpentinite contains enough fluid, and so it usually exhibits low velocity and high attenuation. Previous studies suggest that seismic attenuation is more sensitive than velocity to water content in the crust and upper mantle^21–24^. The low-Q feature of the KM belt may reflect the existence of the serpentinite and high water-content. A significant high-Q anomaly exists beneath the Hidaka metamorphic belt at depths of 10–40 km (Figs 2 and 3), which corresponds to a strong positive Bouguer gravity anomaly striking approximately N-S, reflecting the presence of anomalously high-density rocks, such as mafic and ultramafic rocks in the Hidaka metamorphic belt and the Poroshiri Ophiolite belt^32^. Hence, the high-Q zone reflects the presence of mafic and ultramafic rocks there.

A significant dipping high-Q zone is revealed above the subducting Pacific slab (Figs 2d–g and 3d–g). This high-Q zone exists not only beneath the Ishikari lowland, but also in the southwest of the Ishikari lowland fault system (Figs 2 and 3). This feature was also detected by previous attenuation tomography^16^. Active-source seismic refraction and reflection studies have revealed the northeastward subducting NE Japan arc beneath the Ishikari lowland^6,33^. We think that the dipping high-Q zone represents the subducting NE Japan arc beneath southern Hokkaido.

**Figure 4:**
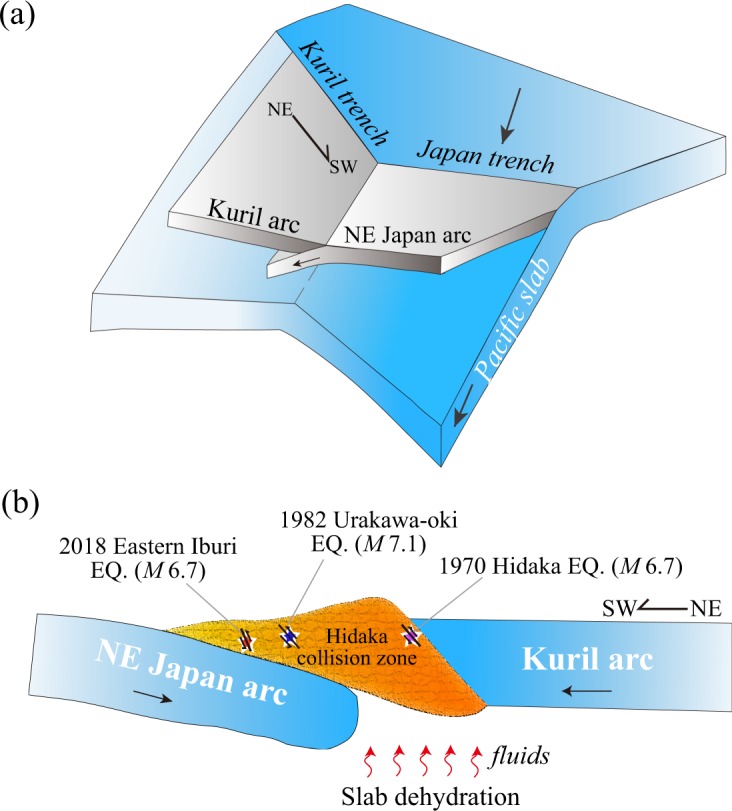
A schematic diagram for the crustal and upper-mantle structure beneath the study region. (**a**) Subduction of the Pacific plate beneath the Okhotsk plate and collision of the Kuril arc with the NE Japan arc. (**b**) A NE-SW vertical cross-section showing the arc-arc collision and mechanism of the three large earthquakes in the Hidaka collision zone. Fluids from dehydration of the subducting Pacific slab also contribute to the generation of the large earthquakes.

A clear low-Qs zone is visible in the forearc mantle wedge beneath the Hidaka collision zone (Fig. 3a,c). The mantle wedge nose is generally characterized by low heat flow^34^ and moderate to weak seismic attenuation (average to higher Q) in the crust and uppermost mantle^15,25,35^, which usually has a low temperature as illustrated by a recent numerical simulation study^36^. In the forearc area, magma cannot be produced because of the low temperature there. The low-Q zone beneath the forearc area may reflect ascending fluids from the slab dehydration, which has been revealed by many previous studies^12,15,16,26,37^. We hence argue that the slab dehydration takes place beneath the forearc region and the resulted fluids ascend to the SY belt and thrust faults in the crust, causing the low-Q zone in the SY belt.

## Discussion

Figure 4 shows a schematic diagram on the generation of the 2018 Eastern Iburi earthquake. Its mainshock and aftershocks occurred at the boundary between high-Q and low-Q zones. As mentioned above, the low-Q zone beneath the SY belt probably reflects the sedimentary rock, high water-content and high crack density^16^. The high-Q zone is located in the southwest of the SY belt and represents part of the subducting NE Japan arc. The Ishikari thrust fault zone extends in the N-S direction, which is located between the low-Q and high-Q zones. The focal mechanism of the 2018 Iburi earthquake (Fig. 1a) shows a reverse fault with a compressional axis in the ENE-WSW direction. Due to the southwestward migration of the Kuril arc and the collision of the Kuril and NE Japan arcs, a series of thrust faults striking in the N-S direction are produced in south-central Hokkaido. The existence of low-Q and low-V zones in the area suggests that the slab dehydration takes place in the forearc and the resulted fluids ascend to the crust. When the fluids enter an active fault in the crust, pore pressure will increase and fault zone friction will decrease, which can trigger large crustal earthquakes (e.g. refs^38–40^).

Whether the 2018 Iburi earthquake was directly associated with the Ishikari lowland fault system or not is one of the fundamental questions^41^. A recent study of the Iburi mainshock and aftershock distribution suggested that the seismogenic fault of this earthquake may be not directly connected with the Ishikari lowland fault system^41^. In the present study, we cannot clarify this issue due to the limited resolution of our Q tomography. However, on the basis of the present findings and previous results, we deem that the 2018 Iburi earthquake was caused by the concentration of stress from the push and squeeze of the NE Japan-Kuril arc collision, as well as overpressure of fluids from dehydration of the subducting Pacific slab. The arc-arc collision caused the unusual focal depth (~37 km) of the Eastern Iburi earthquake.

A previous study^9^ of velocity tomography suggested that the 1970 Hidaka earthquake and the 1982 Urakawa-oki earthquake took place at material boundaries that correspond to the Hidaka thrust fault and the Ishikari fault, respectively. Another study^17^ of velocity tomography revealed a northeastward dipping high-V anomaly beneath the Hidaka belt, which was interpreted as a result of delamination of the NE Japan arc. The delamination of brittle lower crustal material could cause earthquakes with deeper hypocenters than the normal crustal events. However, the western boundary of this high-V anomaly is located at the KM belt, which is far from the epicenter of the 2018 Iburi earthquake. A recent study^42^ of detailed velocity tomography shows that the 2018 Iburi earthquake occurred at the edge of a seismogenic zone with a high P-wave velocity (Vp). This high-Vp zone may reflect a lithospheric fragment and cool down the mantle wedge, which caused the unusually deep hypocenter (~37 km) of the 2018 Iburi earthquake.

The northeastward descending of the NE Japan arc beneath the Kuril arc may have also caused the 1982 Urakawa-oki earthquake (M 7.1), which was a reverse-faulting event with a compressional axis in the NE-SW direction^16,43^. Its focal depth (~26 km) is also deeper than those of normal crustal events (≤~20  km) in the Japanese inland areas^3^. Its hypocenter is also located at the boundary between the low-Q and high-Q zones (Figs 2, 3). As mentioned above, the high-Q zone is interpreted as the subducted NE Japan arc. The study^9^ of velocity tomography beneath southern Hokkaido suggested that the deep hypocenter of the 1982 event was due to a locally lower temperature in the collision zone according to the geothermal gradient data^34^. Our results show that both the 2018 Iburi earthquake and the 1982 Urakawa-oki earthquake occurred at the boundary between the high-Q NE Japan arc and the low-Q SY belt. This boundary is close to the Ishikari lowland fault system. We think that the deep hypocenters of the two large events are attributed to the Kuril- NE Japan arc collision. Compressional stress may be accumulated along the structural boundary (the Ishikari lowland fault system) due to the arc-arc collision and released by the earthquake faulting^43^.

The 1970 Hidaka earthquake (M 6.7) with a focal depth of ~25 km took place in the southern part of the Hidaka collision zone^44^ (Fig. 1). Its hypocenter is also located at a boundary between high-Q and low-Q zones (Figs 2, 3), which corresponds to the Hidaka thrust fault^9,16^. The high-Q zone represents the Hidaka metamorphic belt that belongs to the Kuril arc and exhibits high gravity anomaly^32^, high velocity^9,18,43^ and high electric resistivity^45^. The 1970 Hidaka earthquake had a reverse-faulting mechanism with its fault plane striking in the NW-SE direction^9^. Its occurrence and deep hypocenter (~25 km) may be also caused by the southwestward migration of the Kuril arc^16^.

Thus, all the three large events in southern Hokkaido took place at boundaries between low-Q and high-Q zones. The 1970 Hidaka earthquake occurred on the Hidaka thrust fault, whereas the 1982 Urakawa-oki earthquake and the 2018 Iburi earthquake occurred in the Ishikari lowland fault system. Their focal depths (25–37 km) are all deeper than those of normal crustal events in the Japan Islands^3^, which are mainly controlled by the NE Japan-Kuril arc collision. This tectonic process leads to a SW-NE compressional stress regime and the thrusting of fold and thrust-fault systems over the Hidaka mountain range^46,47^. These large events caused serious hazards to the local society and infrastructure. The generation of the three big earthquakes and high local seismicity in southern Hokkaido indicate that the arc-arc collision is still an ongoing process. The convergence rate between the Eurasian and Okhotsk plates was estimated from the velocity field^48^, which suggested that there is a high potential for a large event in northern Hokkaido. A recent study^49^ of the Coulomb stress change due to the 2018 Iburi earthquake suggested that the Ishikari fault is under increasing seismic threat after the Iburi earthquake. It is expected that large earthquakes would occur again on the Ishikari lowland fault system and the Hidaka thrust fault in the coming decades due to the ongoing arc-arc collision. Hence, we need to pay attention to the seismic risk and prepare for the hazard reduction in the region.

## Conclusion

P and S wave attenuation tomography of the source zone of the 2018 Eastern Iburi earthquake (M 6.7) is determined, which sheds new light on the causal mechanism of the Iburi earthquake that occurred at a boundary between low-Q and high-Q zones. The Sorachi-Yezo belt is imaged as a significant low-Q zone, which may indicate the existence of thick sediments, high water content and high crack density there. A northeastward dipping high-Q zone exists beneath the Ishikari lowland, which is interpreted as the subducted NE Japan arc. Low-Qs anomalies are also revealed in the forearc crust and upper-mantle wedge, which reflect the existence of fluids from dehydration of the subducting Pacific slab. When the fluids enter the active faults in the overlying Okhotsk plate, pore pressure will increase and fault zone friction will decrease, which can trigger a large event such as the 2018 Iburi earthquake. Its unusually deep hypocenter (~37 km) reflects a locally lower temperature due to the northeastward subduction of the NE Japan arc and its collision with the Kuril arc. The 1982 Urakawa-oki earthquake (M 7.1) and the 1970 Hidaka earthquake (M 6.7) were probably caused by the same processes. Similar large earthquakes would occur again in the coming decades on the active thrust faults in southern Hokkaido where the arc-arc collision is an ongoing process. Hence, much attention should be paid to the seismic risk there, and actions should be taken to reduce the seismic hazard.

## Materials and Methods

### Attenuation tomography

The earthquake waveform data used in this study were recorded at 136 permanent seismic stations (Fig. 1b), which belong to the High-sensitivity Seismic Network^50^. The seismograms with a sampling rate of 100 Hz have enough bandwidth for our spectral analysis. We selected 924 local shallow and intermediate-depth earthquakes (*M* > 2.0), which occurred in the study region (Supplementary Fig. S7) during April 2004 to October 2018. These events have reliable hypocentral parameters with mislocation errors smaller than 3 km.

We measured t* data precisely from the P and S wave velocity amplitude spectra of the local earthquake seismograms recorded by the dense seismic network with a frequency range of 0.5–25.0 Hz^51^. The corner frequency of each event is determined using the multi-window spectral ratio method^52^. Supplementary Fig. S1 shows the finally selected 542 events whose corner frequencies are well determined with a root-mean-square residual between the observed and theoretical spectral ratios smaller than 0.3. Examples of the corner frequency determination are shown in Supplementary Fig. S8. After corner frequencies of the local earthquakes are determined, we measure t* by fitting the calculated velocity spectra with the observed ones following the approach of ref.^25^ (Supplementary Fig. S9). As a result, 18,936 t^*^_p_ and 23,066 t^*^_s_ data are measured. The separated steps for estimating the corner frequency and t* enable us to unwarp the trade-off between the two parameters^15,26,37,53^.

The relation between Q and t* is expressed as follows:

1 $${{\rm{t}}}^{\ast }={\int }_{{\rm{ray}}{\rm{path}}}\frac{1}{{\rm{v}}({\rm{s}}){\rm{Q}}({\rm{s}})}{\rm{ds}}$$

where V(s) and Q(s) are seismic velocity and quality factor, respectively, along a ray path *s*. The observation equation can be written as:

2 $${t}_{ij}^{\ast obs}={t}_{ij}^{\ast cal}+{\sum }_{m}\frac{\partial {t}^{\ast }}{\partial {(VQ)}_{m}}\Delta {(VQ)}_{m}+{e}_{ij}$$

where ${t}_{ij}^{\ast obs}$ and ${t}_{ij}^{\ast cal}$ are observed and calculated t* values, respectively, from the *j*th event to the *i*th station, and Δ represents the perturbation of a parameter. A starting 1-D Q model (Supplementary Fig. S10) is firstly determined following the approach of ref.^25^. An initial Q value of 25% higher than the 1-D Q model is assigned to the subducting Pacific slab, because the slab is found to have a low attenuation (i.e., high Q) by previous studies^25,35,54^. Following the previous tomographic studies of the Japan subduction zone^15,25,26,55,56^, we set up two 3-D grids with a lateral grid interval of 0.2° in the study volume. One grid is for parameterizing the crust and upper mantle, whereas the other grid is for parameterizing the subducting Pacific slab. Meshes of grid nodes are set at depths of 10, 25, 40, 65, 90, 140, 190 and 240 km in the crust and mantle. The value of a parameter (such as *V*, *Q* or *VQ*) at any point in the study volume is calculated from its values at eight grid nodes surrounding that point using a linear interpolation scheme^55^. An efficient 3-D ray tracing technique^55^ is used to compute theoretical t* and ray paths precisely. In the Q tomographic inversion, lateral depth variations of the Conrad and Moho discontinuities are also taken into account. The damped least-squares method^55^ is used to solve the large but sparse system of observation equations that relate the observed t* to the unknown *VQ* parameters. Dividing *VQ* by V at each grid node, we can obtain a 3-D *Q* model. The 3-D velocity (*Vp*, *Vs*) model used in this work is the high-resolution model of the Japan subduction zone^57^. Supplementary Fig. S11 shows trade-off curves that are constructed for determining the optimal values of the damping and smoothing parameters for the Qp and Qs tomography. The root-mean-square (RMS) ${{\rm{t}}}_{{\rm{p}}}^{\ast }$ residuals before and after the 3-D inversion are 0.0238 and 0.0204, and the corresponding RMS ${{\rm{t}}}_{{\rm{s}}}^{\ast }$ residuals are 0.0279 and 0.0234, respectively. For details of the method, see refs^15,25^.

### Resolution tests

Our study volume is well covered by the ray paths, especially in and around the 2018 Iburi earthquake area (Supplementary Fig. S12). To further evaluate the adequacy of the ray coverage and to assess the spatial resolution of the tomographic images, we conducted extensive checkerboard resolution tests (CRTs) with a lateral interval of 0.2° ^25,55,56^. To perform a CRT, we first assign positive and negative Q perturbations of 40% at the adjacent grid nodes in the input model to calculate the synthetic t*. Then Gaussian noise with a standard deviation of 0.002 (about 10% of the RMS residuals) is added to the synthetic t* before performing the tomographic inversion. Supplementary Fig. S5 shows the obtained CRT results with a lateral interval of 0.2°, which indicate that the input model can be well recovered. The CRT results are consistent with the distribution of seismic stations and earthquakes used in this study (Fig. 1b and Supplementary Fig. S1). To further evaluate the robustness of the obtained 3-D Qp and Qs models, we conducted restoring resolution tests (RRTs)^55^. The procedure of the RRT is the same as that of the CRT, except for the input model that is derived from the obtained Q tomographic results (Figs 2 and 3). The RRT results (Supplementary Fig. S6) show that main features of the Qp and Qs tomography can be well recovered, suggesting that the 3-D attenuation results are robust.

## Supplementary information

Supporting information

## Data Availability

All data needed to evaluate the conclusions in the paper are present in the paper and/or the Supplementary Materials. Additional data related to this paper may be requested from the authors.
