# Supplementary material for: Arc-arc collision caused the 2018 Eastern Iburi earthquake (M 6.7) in Hokkaido, Japan
Source: Sci Rep. 2019 Sep 26;9:13914. doi: 10.1038/s41598-019-50305-x (PMC6763458; doi:10.1038/s41598-019-50305-x)
Supplement: Supplementary file 1 — Supporting information [file 41598_2019_50305_MOESM1_ESM.pdf]

Supporting information for

Arc-arc collision caused the 2018 Eastern Iburi earthquake (*M* 6.7) in Hokkaido, Japan

Yuanyuan Hua[1,2*], Dapeng Zhao[1*], Yixian Xu[3], Zewei Wang[1,4]

1. Department of Geophysics, Graduate School of Science, Tohoku University, Sendai 980-8578, Japan
2. Subsurface Multi-scale Imaging Key Laboratory of Hubei Province, Institute of Geophysics and Geomatics, China University of Geosciences, Wuhan 430074, China
3. School of Earth Sciences, Zhejiang University, Hangzhou, Zhejiang 310027, China
4. School of Earth Sciences and Engineering, Sun Yat-sen University, Guangzhou, China

Submitted to ***Scientific Reports*** in February 2019

Revised version submitted in August 2019

***Corresponding authors.**

Yuanyuan Hua (huayy2016@gmail.com)

Dapeng Zhao (zhao@tohoku.ac.jp)

Contents of this file

Figures S1 to S12

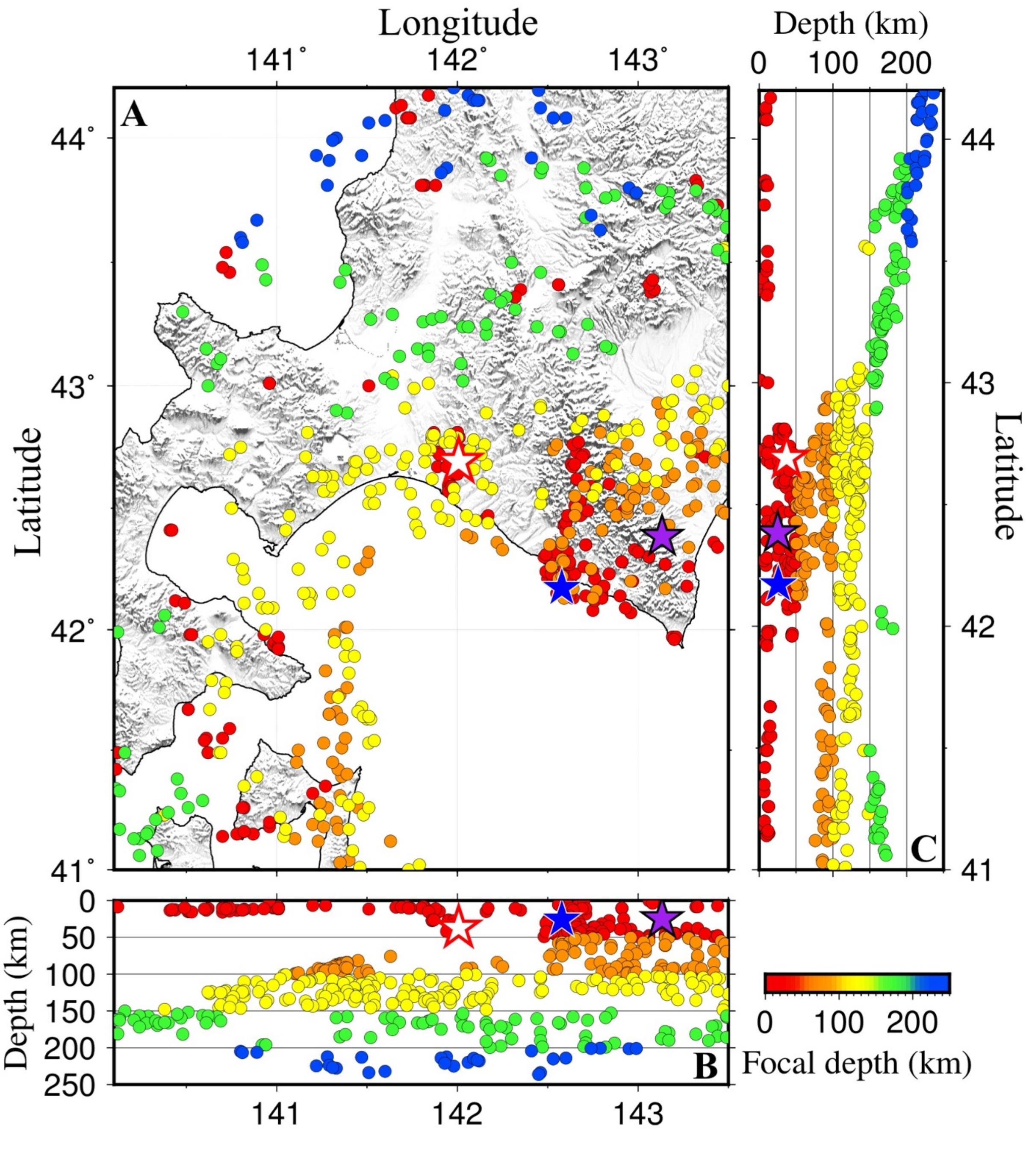

Fig. S1. Local earthquakes used in this study. (**A**) Map view showing the epicentral distribution of 542 local shallow and intermediate-depth earthquakes used in this study. (**B**) East-west and (**C**) north-south vertical cross-sections of the 542 earthquakes shown in (**A**). The red, blue and purple stars denote the 2018 Eastern Iburi earthquake (M 6.7), the 1982 Urakawa-oki earthquake (M 7.1) and the 1970 Hidaka earthquake (M 6.7), respectively. This figure was generated using the Generic Mapping Tools[1] version 5.4.3 (http://gmt.soest.hawaii.edu).

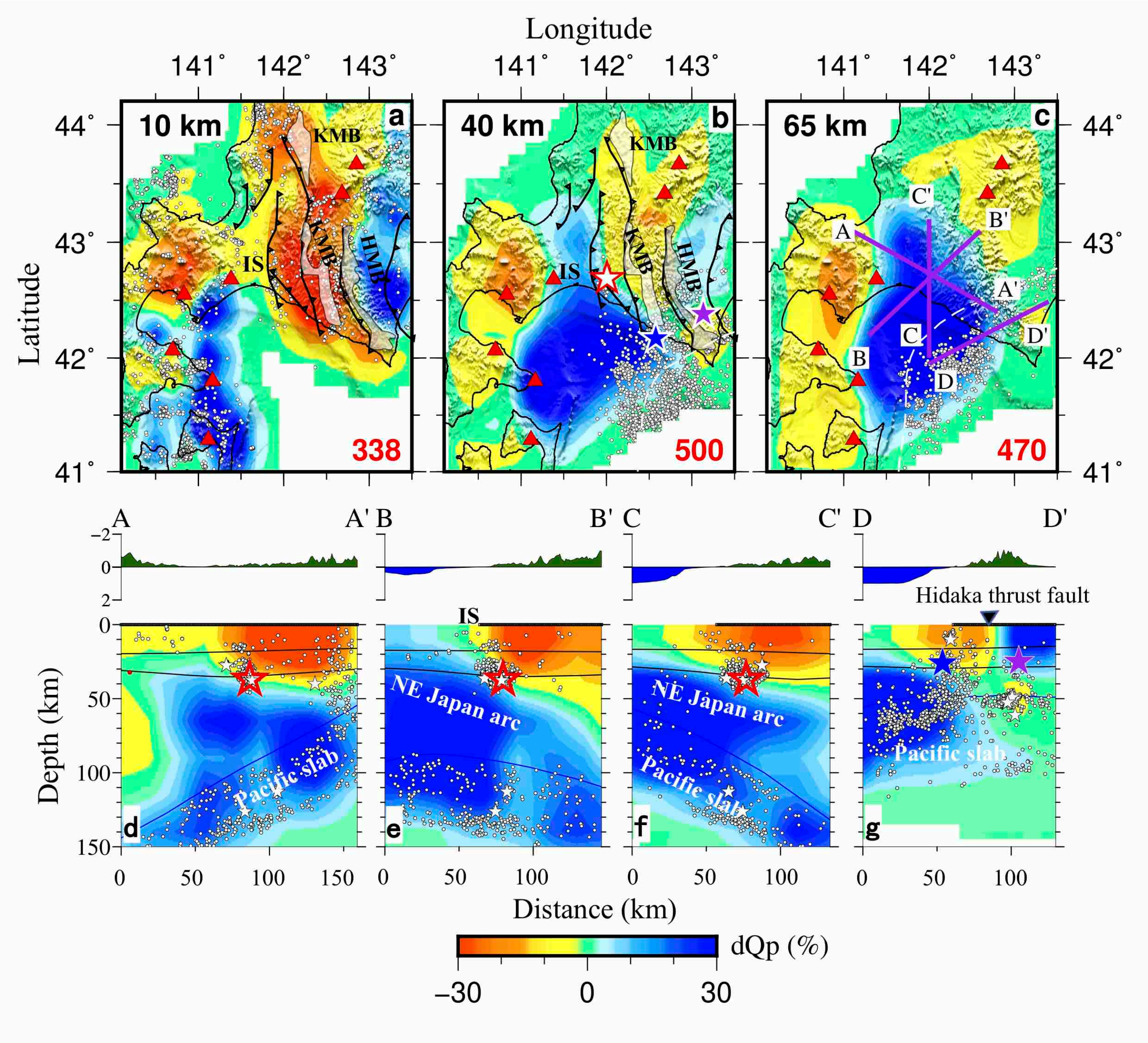

Fig. S2. Results of P-wave attenuation tomography without the predefinition of the high-Q subducting slab. The same as Fig. 2 but for tomographic inversions without the predefinition of the low-attenuation (high-Q) subducting Pacific slab. This figure was generated using the Generic Mapping Tools[1] version 5.4.3 (http://gmt.soest.hawaii.edu).

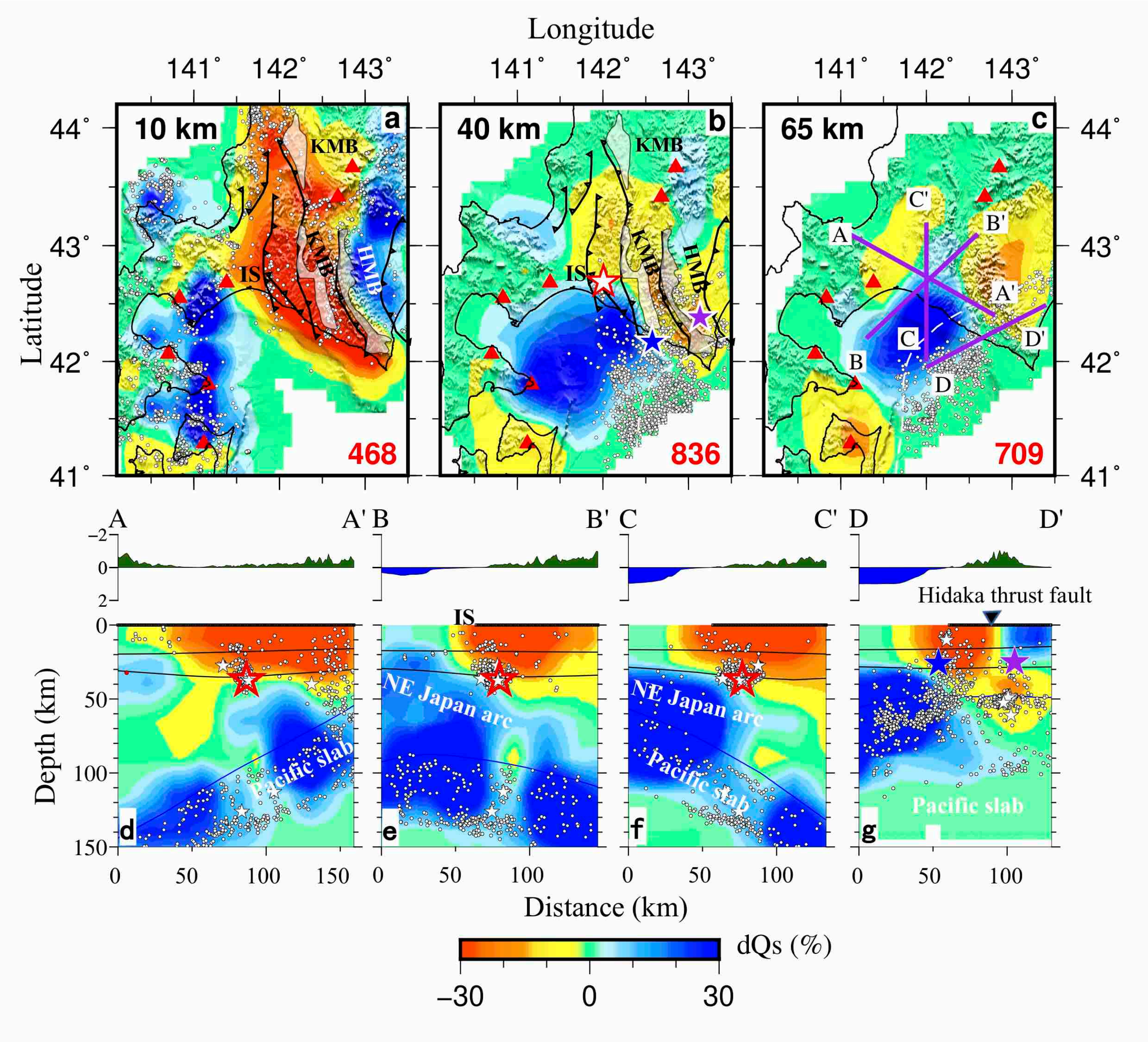

Fig. S3. Results of S-wave attenuation tomography without the predefinition of the high-Q subducting slab. The same as Fig. 3 but for tomographic inversions without the predefinition of the low-attenuation (high-Q) subducting Pacific slab. This figure was generated using the Generic Mapping Tools[1] version 5.4.3 (http://gmt.soest.hawaii.edu).

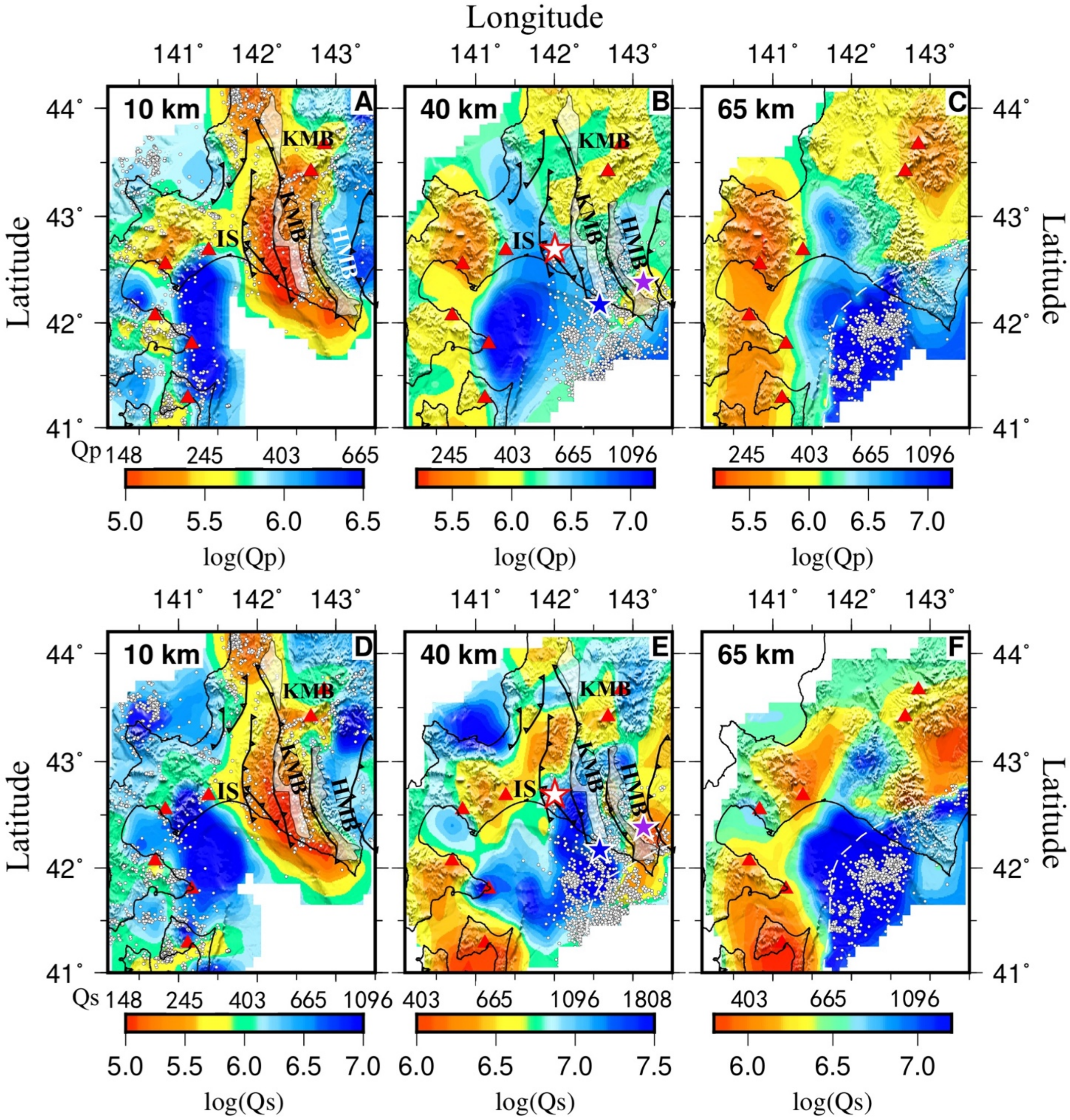

Fig. S4. P and S wave attenuation tomography obtained by using the L-BFGS-B method. (A to C) denote map views of P-wave attenuation (Qp) tomography at three depths. The layer depth is shown at the upper-left corner of each map. The red and blue colors denote low and high Q values, respectively, whose scale is shown below each map. (D to F) are the same as (A to C) but for S-wave attenuation (Qs) tomography. This figure was generated using the Generic Mapping Tools[1] version 5.4.3 (http://gmt.soest.hawaii.edu).

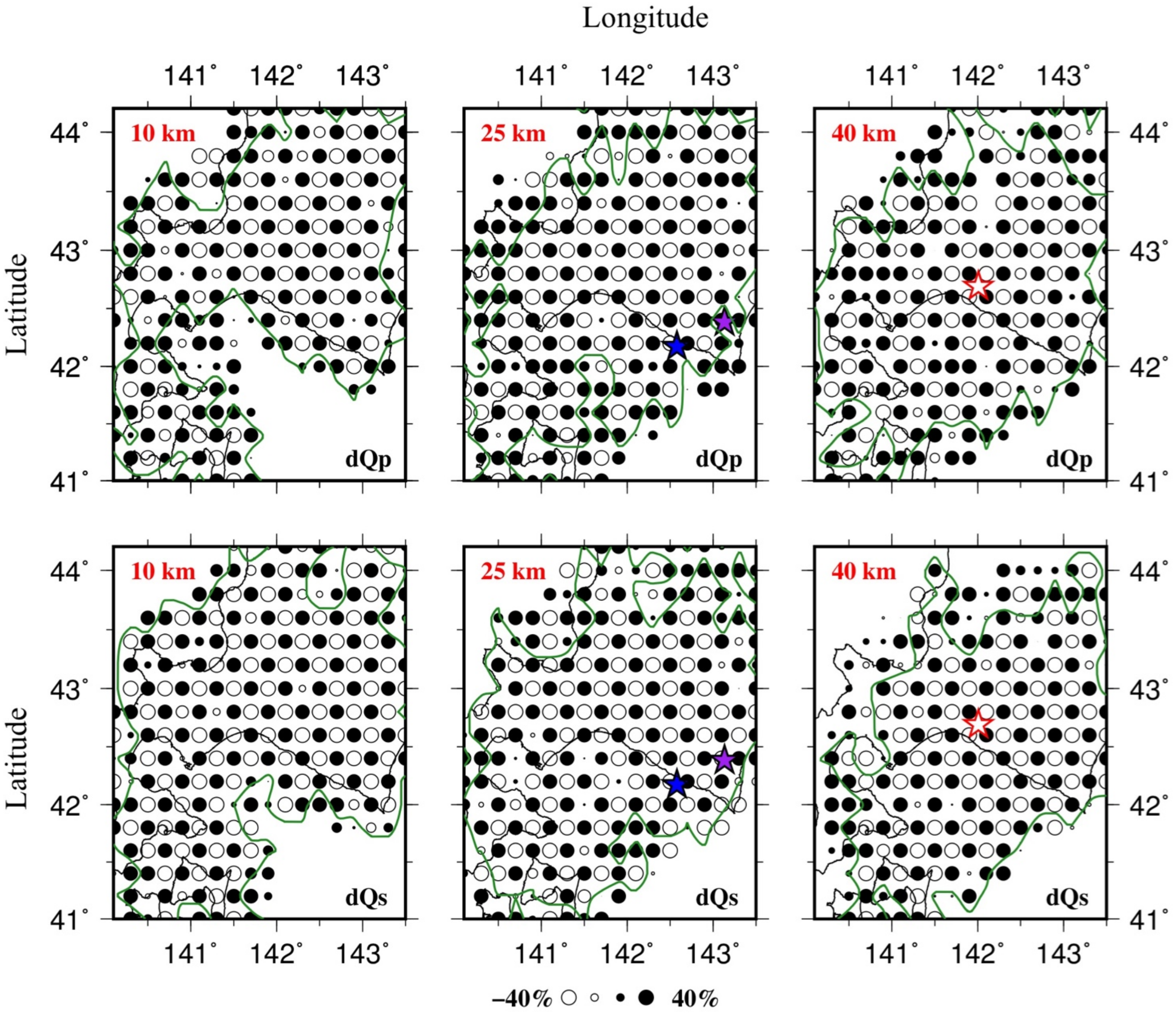

Fig. S5. Results of checkerboard resolution tests (CRTs) for P and S wave attenuation tomography. The lateral grid interval is 0.2°. The solid and open circles denote high and low Q perturbations, respectively, whose scale is shown at the bottom. The red, blue and purple stars denote the 2018 Eastern Iburi earthquake (M 6.7), the 1982 Urakawa-oki earthquake (M 7.1) and the 1970 Hidaka earthquake (M 6.7), respectively. The well-resolved area is outlined by green lines. This figure was generated using the Generic Mapping Tools[1] version 5.4.3 (http://gmt.soest.hawaii.edu).

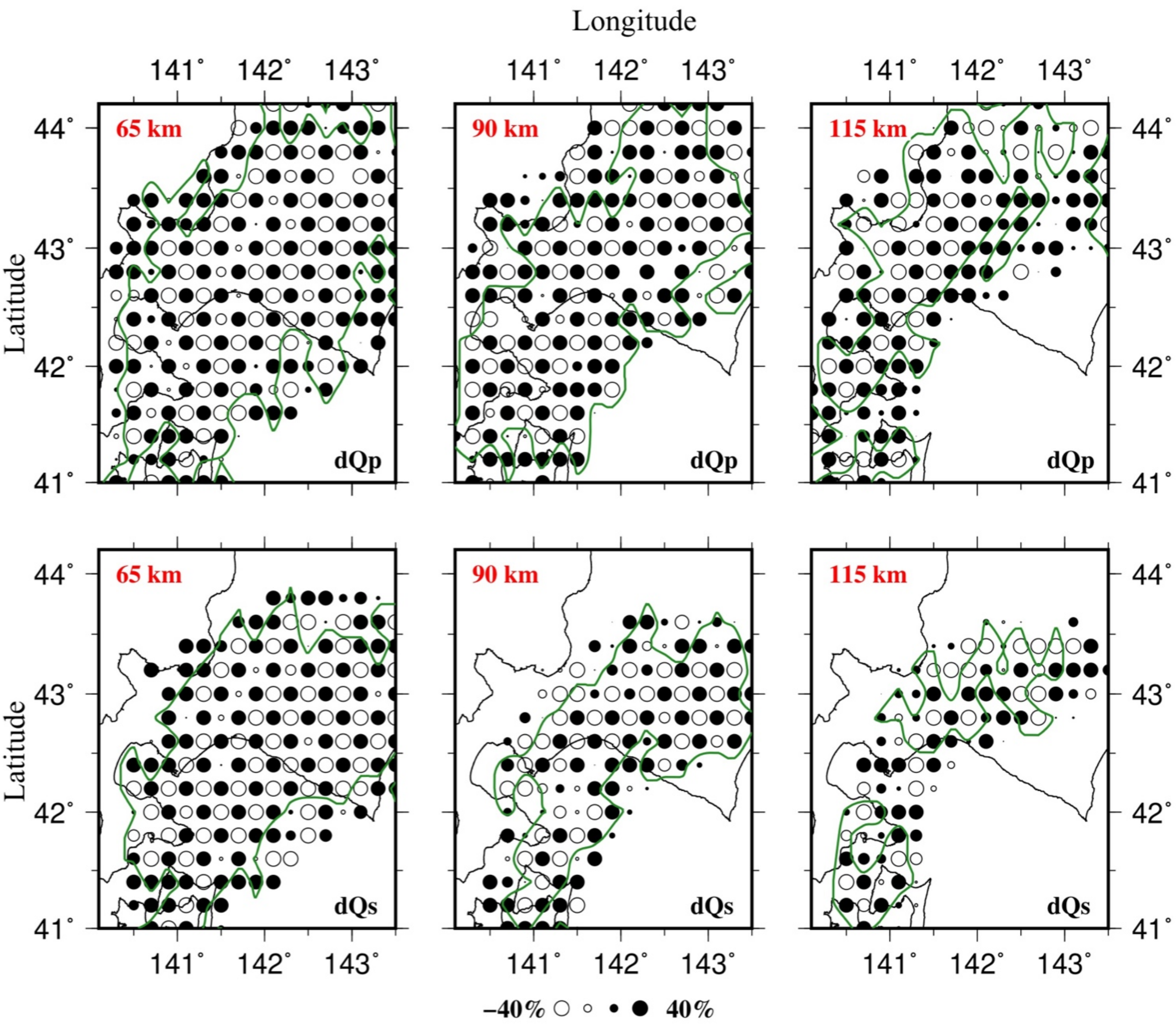

Fig. S5. (continued)

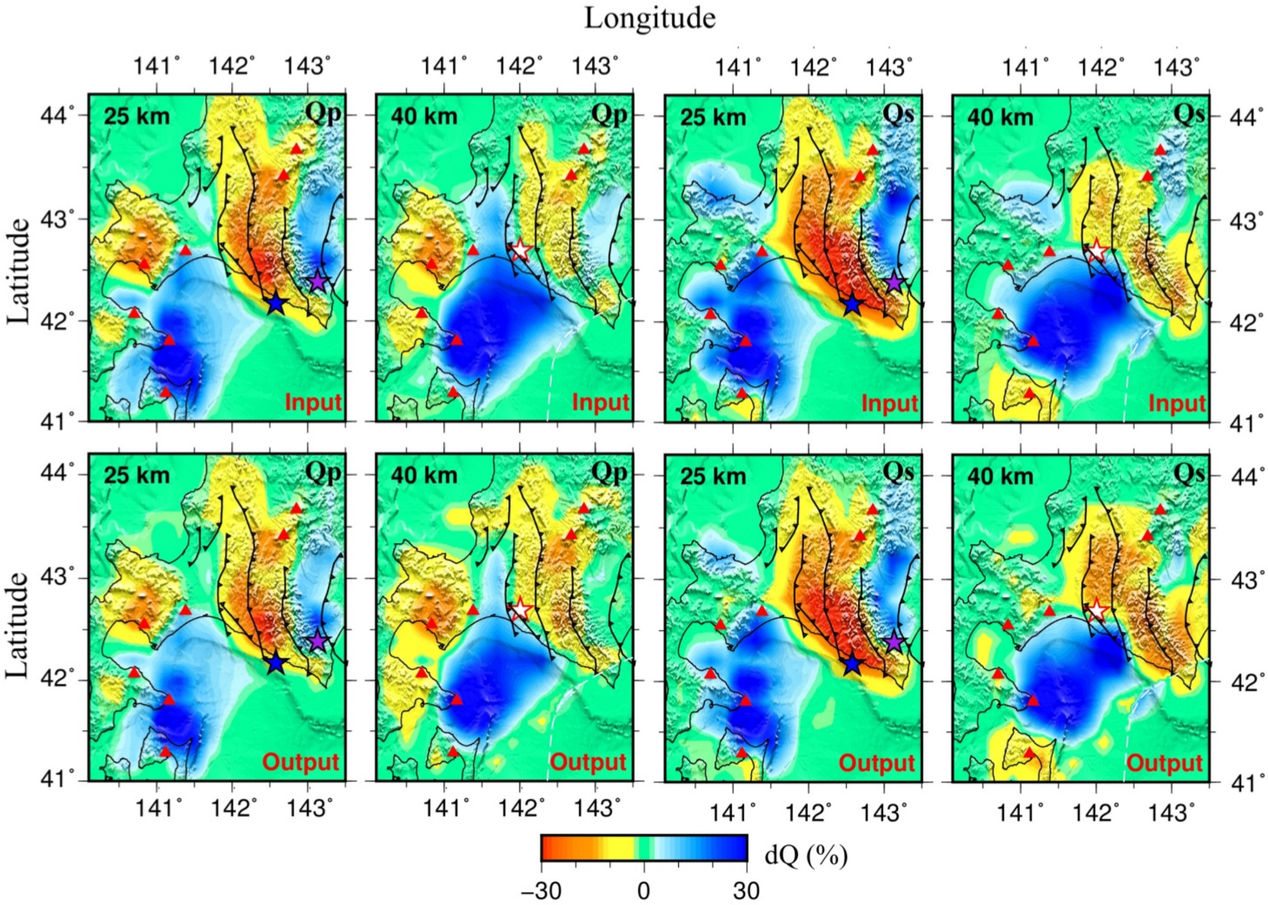

Fig. S6. Results of restoring resolution tests (RRTs) for P and S wave attenuation tomography. The obtained 3-D Q model is taken to be the input model, which is shown in the upper panels. Gaussian random noise with a standard deviation of 0.002 s (corresponding to the data error) is added to the theoretical t* before conducting the attenuation tomography. The lower panels show the RRT results. The red and blue colors denote low and high Q perturbations, respectively, whose scale is shown at the bottom. The red, blue and purple stars denote the 2018 Eastern Iburi earthquake (M 6.7), the 1982 Urakawa-oki earthquake (M 7.1) and the 1970 Hidaka earthquake (M 6.7), respectively. This figure was generated using the Generic Mapping Tools[1] version 5.4.3 (http://gmt.soest.hawaii.edu).

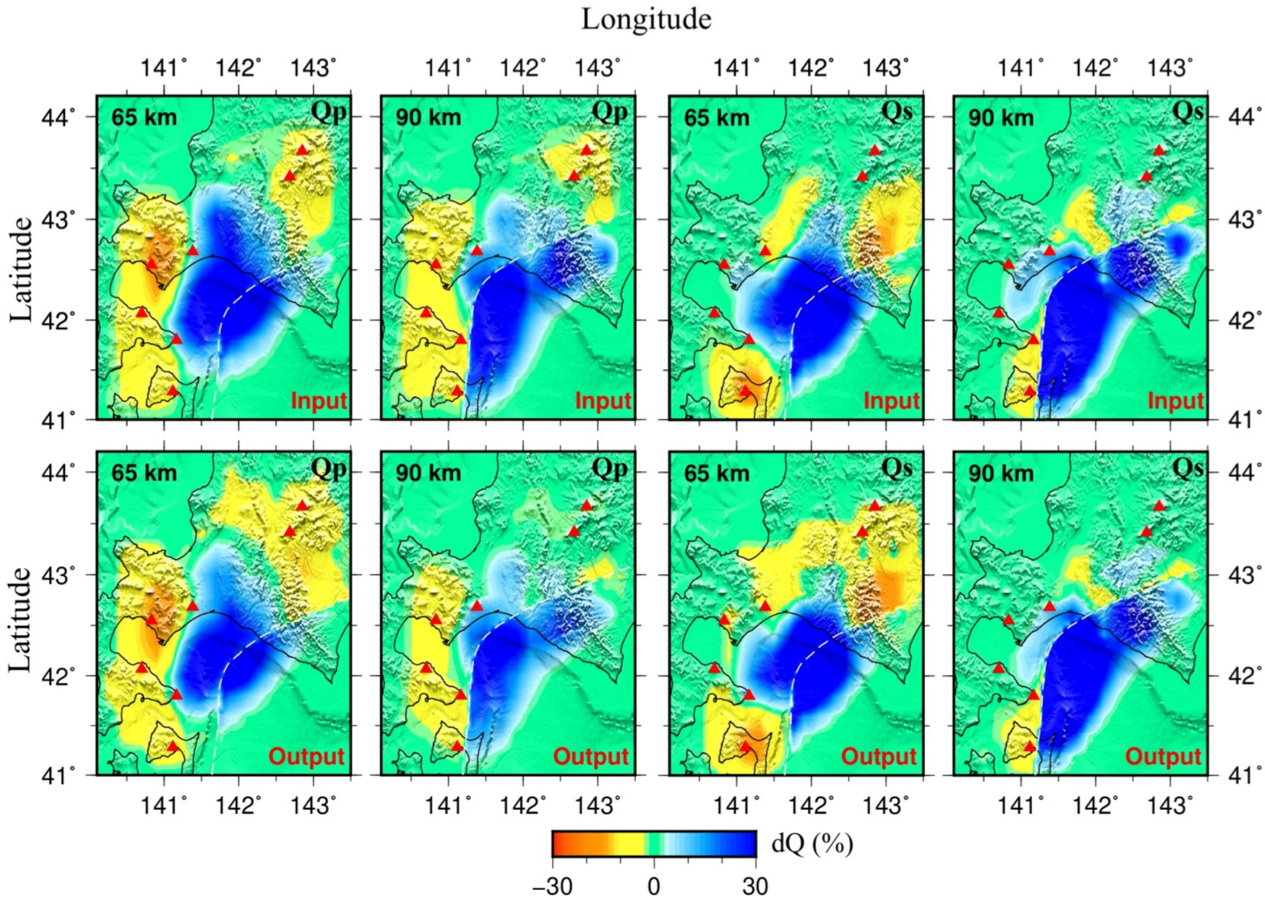

Fig. S6. (continued)

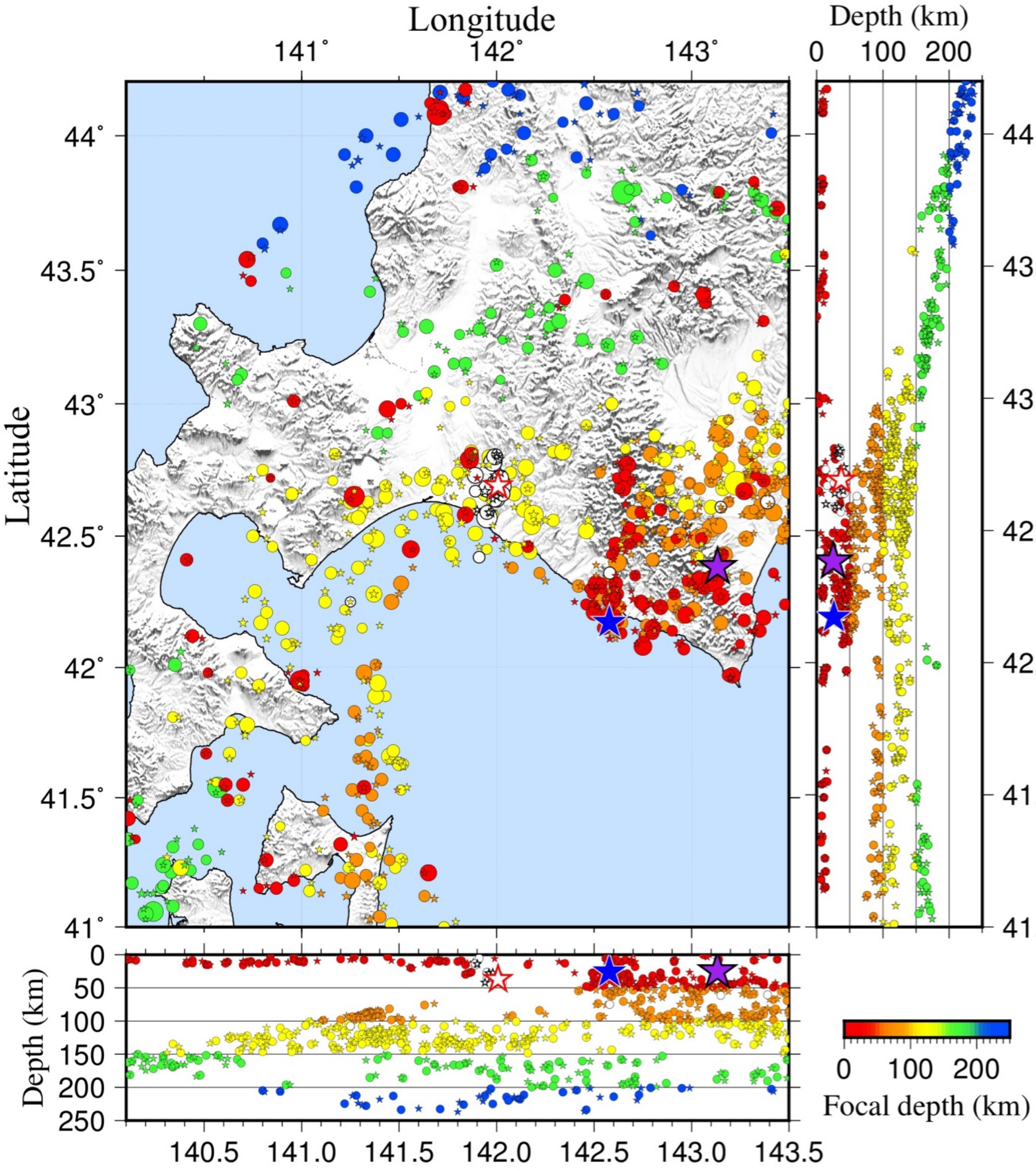

Fig. S7. Distribution of 462 event pairs used in this study. The circles and stars denote large and small earthquakes, respectively. Colors of the symbols denote focal depths whose scale is shown at the lower-right corner. The big red, blue and purple stars denote the 2018 Eastern Iburi earthquake (M 6.7), the 1982 Urakawa-oki earthquake (M 7.1) and the 1970 Hidaka earthquake (M 6.7), respectively. This figure was generated using the Generic Mapping Tools[1] version 5.4.3 (http://gmt.soest.hawaii.edu).

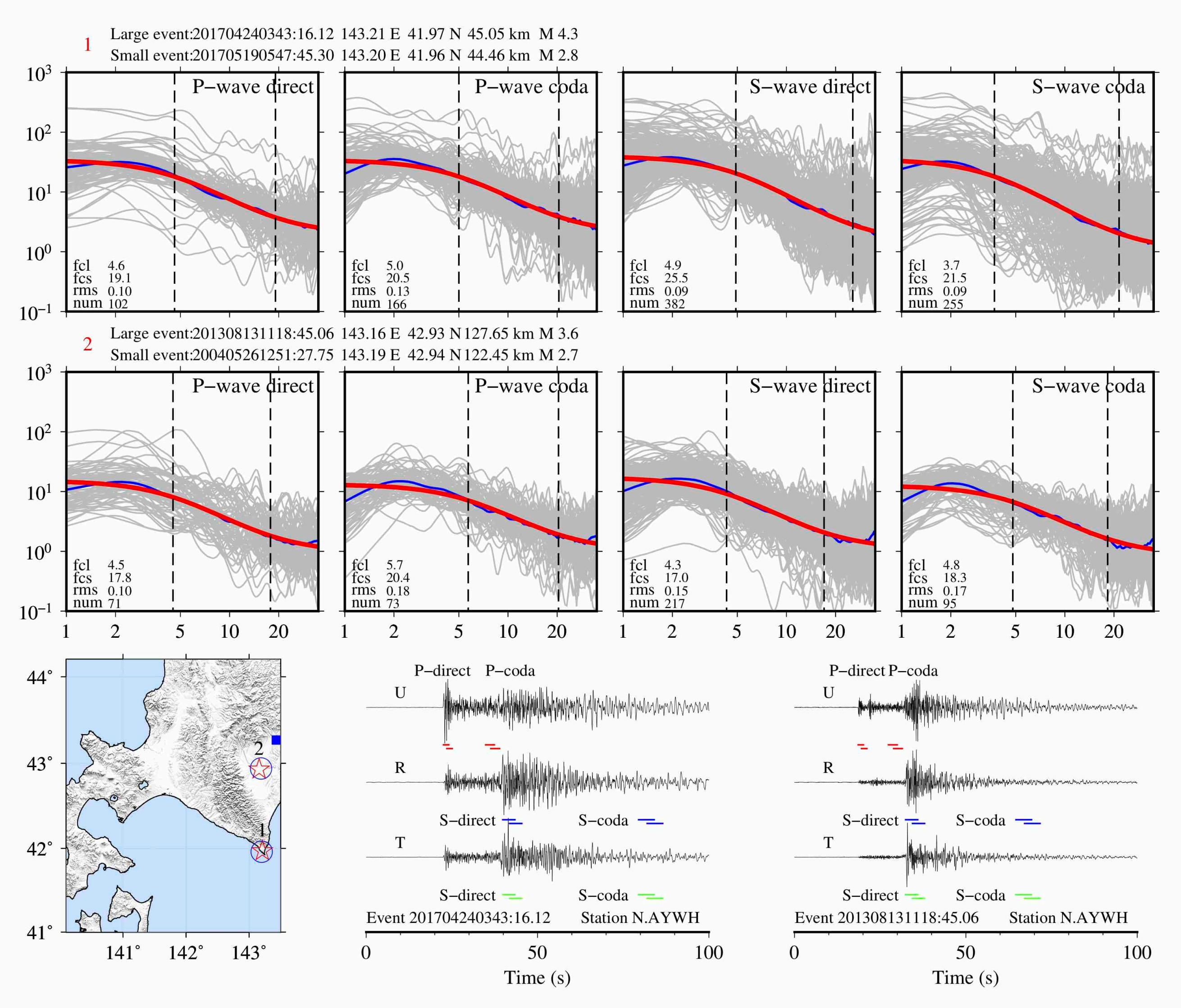

Fig. S8. Examples of multi-window spectral ratio analysis for two event pairs. In the upper eight panels, the gray thin lines denote spectral ratios of seismograms in different time windows, components and stations. The red and blue lines denote the calculated and stacked spectral ratios, respectively. The two vertical dotted lines denote corner frequencies of large and small events. The corner frequencies of large and small events (fcl, fcs), the root-mean-square (rms) of spectral ratio residuals and the number (num) of spectral ratios used to determine the stacked spectral ratios are shown at the bottom-left of each panel. The hypocentral parameters of each event pair are shown above the spectral ratio panels. Epicenters of the two event pairs are shown in the inset map. Up-down (U), radial (R) and tangential (T) component seismograms of the two larger events recorded at station N.AYWH are shown beside the inset map. The different time windows are shown in red, blue and green lines below the seismograms. This figure was generated using the Generic

Mapping Tools[1] version 5.4.3 (http://gmt.soest.hawaii.edu).

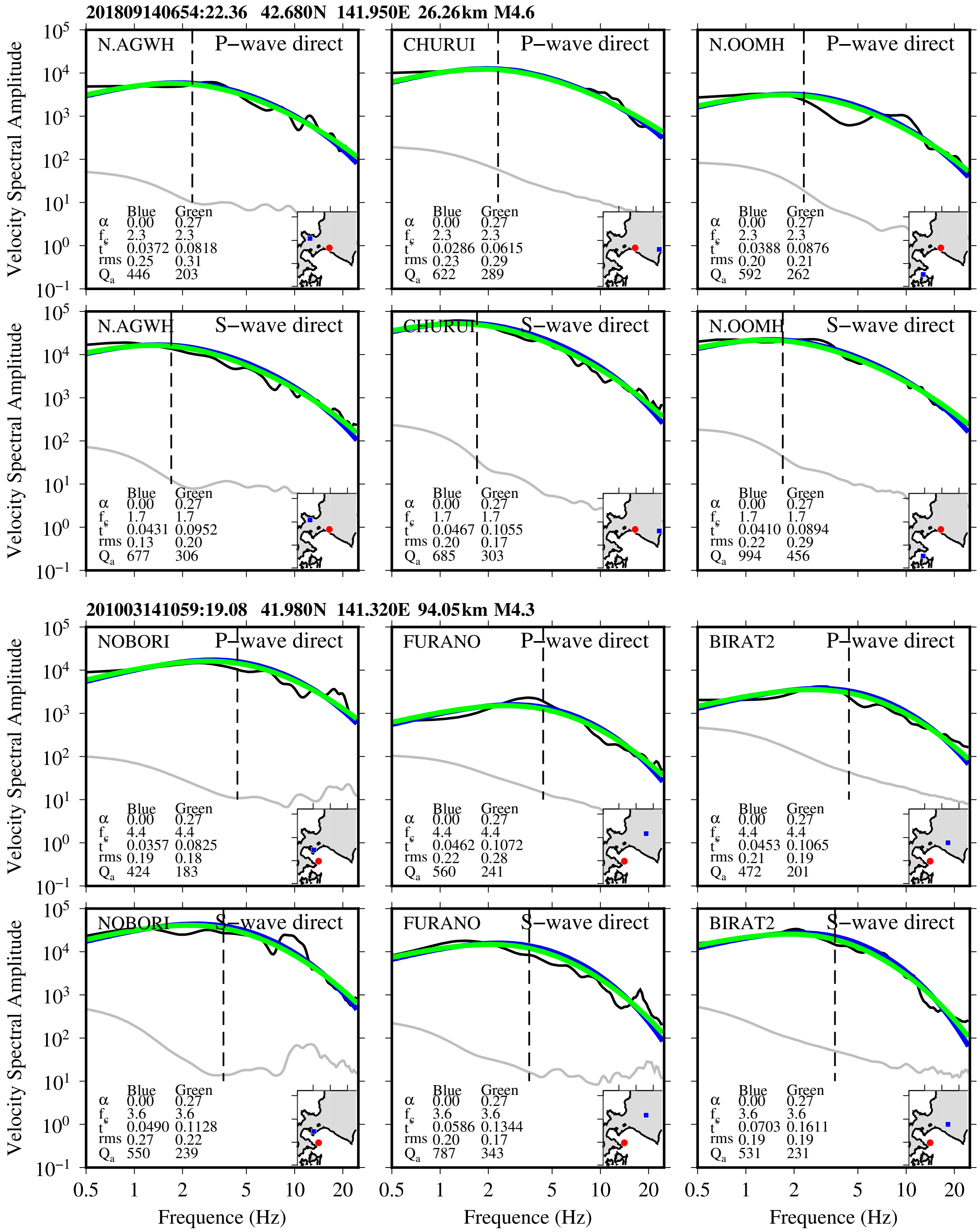

Fig. S9. Examples of measuring t* for two earthquakes recorded at different stations. In each panel, the gray and black lines denote the noise and signal spectra, respectively. The blue and green lines represent the calculated frequency-independent (α = 0) and frequency-dependent (α = 0.27)

spectra, respectively. The vertical dashed line denotes the corner frequency of each event. This figure was generated using the Generic Mapping Tools[1] version 5.4.3 (http://gmt.soest.hawaii.edu).

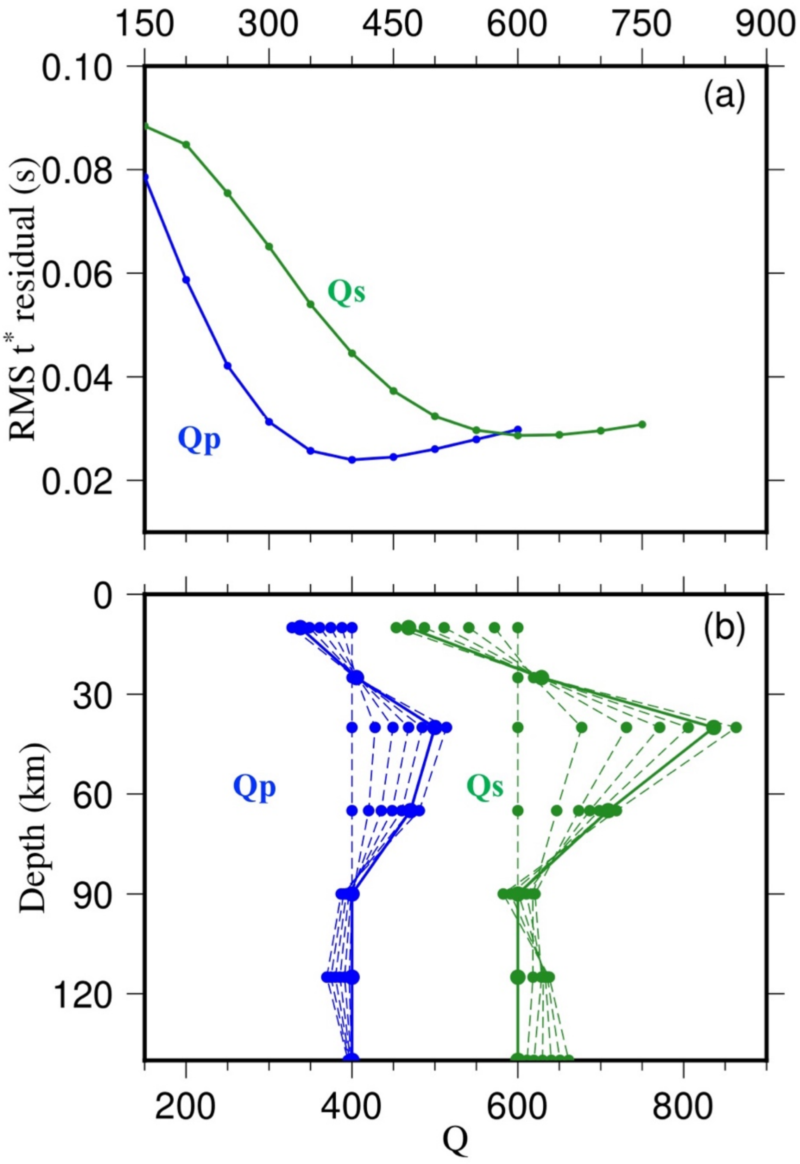

Fig. S10. 1-D Q model used in this study. (a) Relationship between the uniform Q values at all depths and the corresponding root-mean-square (RMS) t* residuals. (b) The dashed lines denote average 1-D Q models obtained after many iterations. The solid lines are the optimal 1-D *Qp* (blue) and *Qs* (green) models. This figure was generated using the Generic Mapping Tools[1] version 5.4.3 (http://gmt.soest.hawaii.edu).

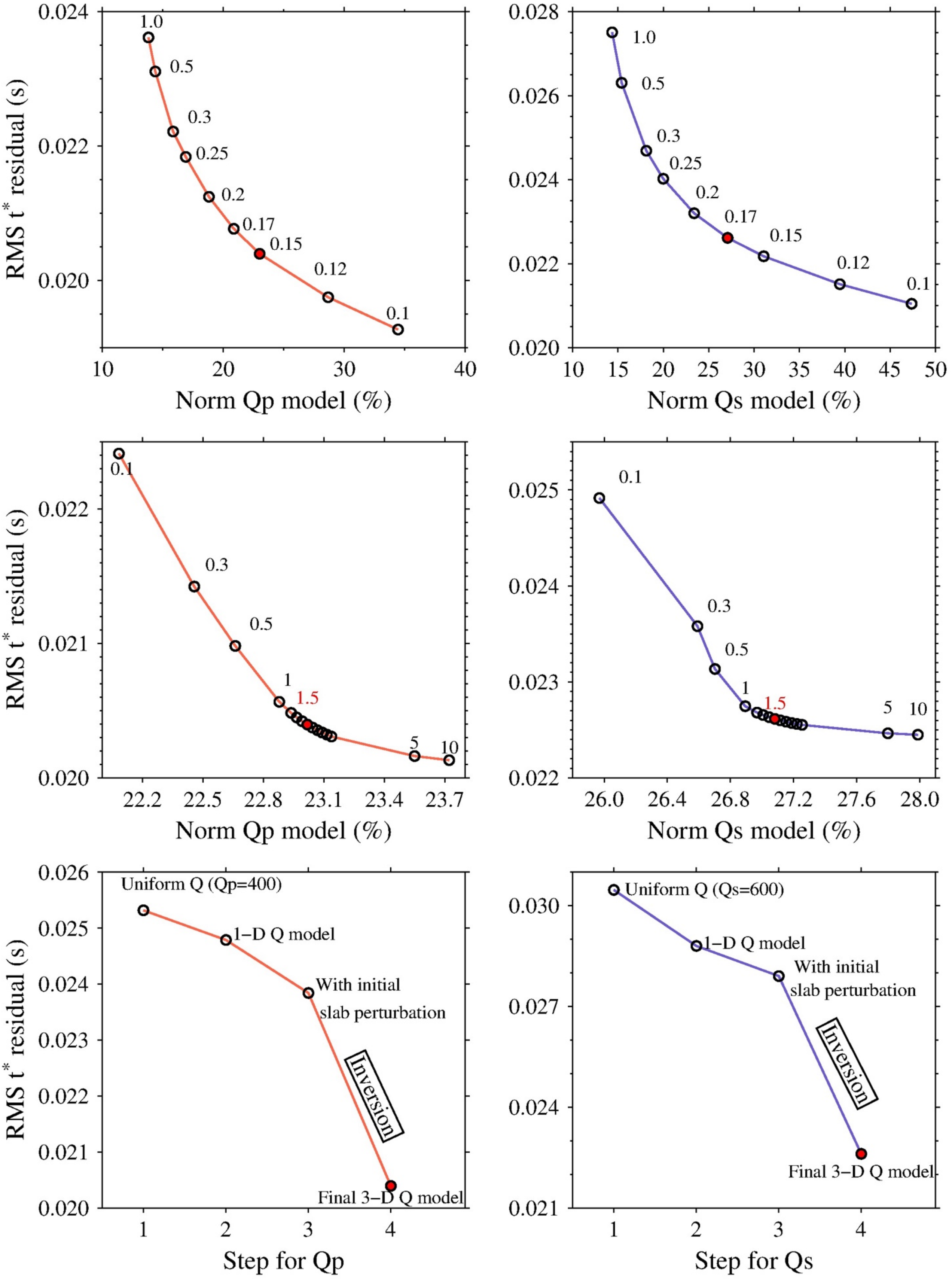

Fig. S11. Trade-off curves and RMS residuals for the tomographic inversions. Trade-off curves for determining the optimal damping parameters for Qp (a) and Qs (b) tomography. (c, d) Trade-off

curves for determining the smoothing parameters for Qp and Qs, respectively. The numbers beside the open circles are different values of the damping and smoothing parameters, whereas those beside the solid red dots show the optimal values. (e, f) Reductions of the RMS t* residuals for Qp and Qs tomography, respectively. Step 1: searching for an optimal uniform Q model; Step 2: determining the final 1-D Q models; Step 3: adding an initial Q perturbation to the subducting Pacific slab; Step 4: conducting the 3-D Q inversion. This figure was generated using the Generic Mapping Tools[1] version 5.4.3 (http://gmt.soest.hawaii.edu).

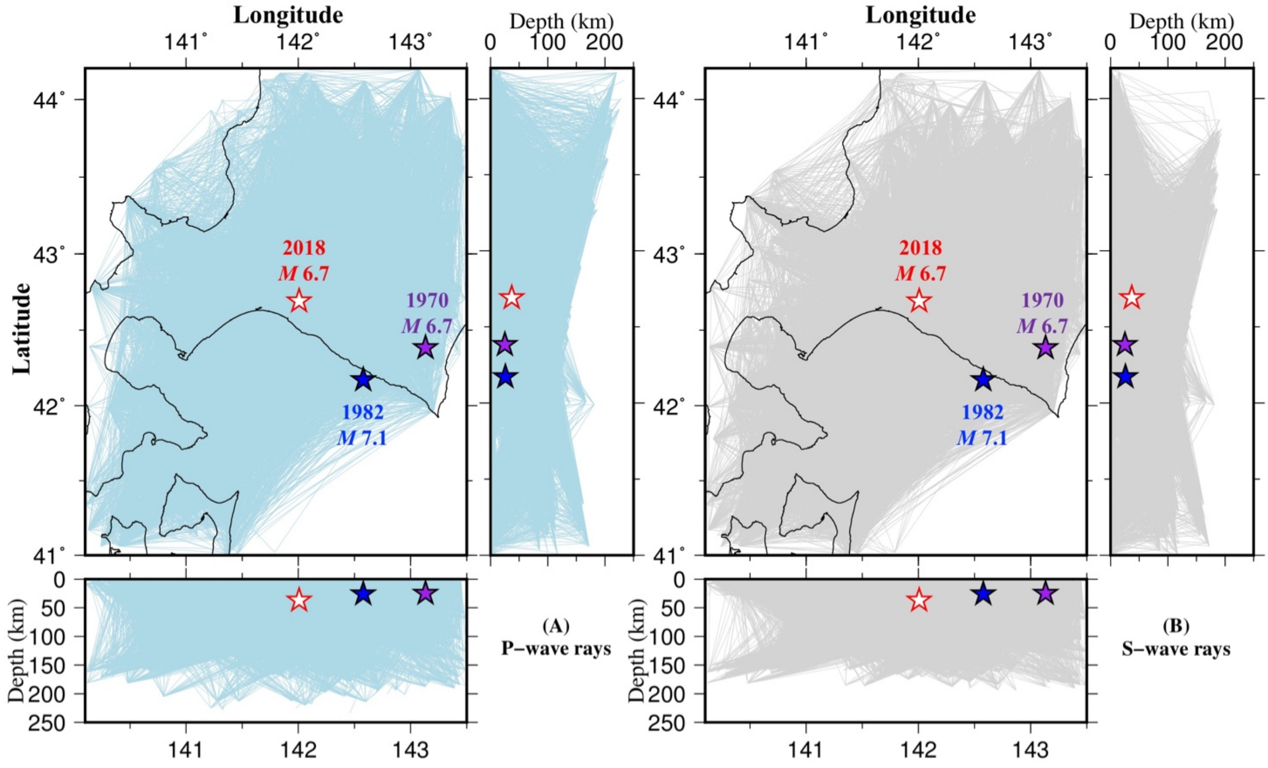

Fig. S12. Distribution of ray paths in the study region. (A) P and (B) S wave rays used in this study. The red, blue and purple stars denote the 2018 Eastern Iburi earthquake (M 6.7), the 1982 Urakawa-oki earthquake (M 7.1) and the 1970 Hidaka earthquake (M 6.7), respectively. This figure was generated using the Generic Mapping Tools[1] version 5.4.3 (http://gmt.soest.hawaii.edu).

References

1. Wessel, P., Smith, W. H. F., Scharroo, R., Luis, J. & Wobbe, F. Generic Mapping Tools: Improved Version Released. *Eos, Transactions American Geophysical Union* **94**, 409–410 (2013).
